# Perceptual Training Prevents the Emergence of the Other Race Effect during Infancy

**DOI:** 10.1371/journal.pone.0019858

**Published:** 2011-05-18

**Authors:** Michelle Heron-Delaney, Gizelle Anzures, Jane S. Herbert, Paul C. Quinn, Alan M. Slater, James W. Tanaka, Kang Lee, Olivier Pascalis

**Affiliations:** 1 Department of Psychology, University of Sheffield, Sheffield, United Kingdom; 2 Department of Human Development and Applied Psychology, University of Toronto, Toronto, Ontario, Canada; 3 Department of Psychology, University of Delaware, Newark, Delaware, United States of America; 4 Psychology Department, University of Exeter, Exeter, United Kingdom; 5 Department of Psychology, University of Victoria, Victoria, British Columbia, Canada; 6 Institute of Child Study, University of Toronto, Toronto, Ontario, Canada; 7 Laboratoire de Psychologie et Neurocognition, Université Pierre Mendes France, Grenoble, France; French National Centre for Scientific Research, France

## Abstract

Experience plays a crucial role in the development of the face processing system. At 6 months of age infants can discriminate individual faces from their own and other races. By 9 months of age this ability to process other-race faces is typically lost, due to minimal experience with other-race faces, and vast exposure to own-race faces, for which infants come to manifest expertise [1]. This is known as the Other Race Effect. In the current study, we demonstrate that exposing Caucasian infants to Chinese faces through perceptual training via picture books for a total of one hour between 6 and 9 months allows Caucasian infants to maintain the ability to discriminate Chinese faces at 9 months of age. The development of the processing of face race can be modified by training, highlighting the importance of early experience in shaping the face representation.

## Introduction

The Other Race Effect (ORE) reflects a difficulty in recognizing and discriminating between faces from other-race groups compared to faces of one's own race, and is a well-established phenomenon in adults (see [2] for a review). The ORE is assumed to be a consequence of experience with faces from races that are typically found in our environment. Thus, extensive experience with one's own race results in expertise in identifying individuals within that group, whereas lack of experience with other-race individuals results in relatively poor discrimination and recognition abilities for these faces.

Recent research on the developmental origins of the ORE has revealed that sensitivity to race emerges early in life. This research supports the perceptual narrowing hypothesis that face representation at birth is broad and develops according to the type of facial input received [3]. For example, facial input received by infants during the first 3 months of life is sufficient to drive a visual preference for own-race faces [4], [5], [6]. Kelly and colleagues [4] have shown that this preference is not present at birth in Caucasian infants and Bar-Haim et al. [5] found that 3-month-old Africans exposed to Caucasian and African faces did not show a preference for either kind of face. Thus, infants' preference for different faces is dependent on their experience with faces of a given race.

This early visual preference for familiar categories of faces in the visual environment may be the starting point for the development of the ORE. Kelly et al. [1] assessed Caucasian 3-, 6-, and 9-month-olds' abilities to discriminate own-race faces and other-race African, Middle Eastern, and Chinese faces. Three-month-olds demonstrated recognition of faces in all four races, whereas 9-month-olds only recognized own-race (Caucasian) faces. A similar study with Chinese infants [7] reported a comparable developmental trajectory, with recognition limited to Chinese faces by 9 months of age. These findings suggest that facial input from the infant's environment is fundamental in shaping the face-processing system in infancy, and that the ORE is firmly in place by 9 months of age [1].

Other studies also reveal a difference in face processing abilities for own- and other-race faces that emerges by approximately 9 months. Ferguson, Kulkofsky, Cashon and Casasola [8] found a difference in the way Caucasian infants process own versus other-race faces for 8-, but not 4-month-olds. At 4 months, Caucasian infants process both own- and other-race faces holistically. However, by 8 months infants process own-race faces holistically, and other-race faces featurally, suggesting that narrowing in processing, as well as discrimination abilities, is occurring at roughly the same ages [8].

Although the face-processing system becomes tuned to own-race faces very early in life, it still retains flexibility to process other-race faces, given sufficient training or exposure. A fragile ORE in 3-month-old Caucasians was abolished by exposing the infants to three Asian faces (i.e., rather than one) during the learning phase [9]. It appears that a robust ORE does not emerge until later in life. However, even after a robust ORE has emerged in childhood, the face-processing system remains flexible so that the ORE can be removed or even reversed if there is sufficient exposure to other-race faces [10], [11].

Whilst the ORE has been consistently demonstrated in adults, it has been shown to be reducible, at least in the short term, with minimal training. Training participants to discriminate other-race faces can increase their ability to discriminate and recognize other-race faces and reduce the ORE [12], [13], [14]. In particular, experience individuating other-race faces (e.g. Joe, Bob) rather than categorizing faces (Chinese vs. African) improves recognition performance [15], [16]. Results from these training studies suggest that the recognition of other-race faces in adulthood is still flexible and responsive to perceptual experience.

The aforementioned studies demonstrate that the ORE can be reduced in children and adults given sufficient exposure to other-race faces. The present study will ascertain if the ORE can be prevented from developing in the first place if infants are given perceptual experience with other-race faces following a training methodology that has been used to maintain infants' abilities to discriminate among faces from another species (Pascalis et al., 2005). Prior research indicates that 6-month-olds can discriminate both human and monkey faces, whereas 9-month-olds and adults can only discriminate human faces due to perceptual narrowing of the face representation [17]. In Pascalis et al. [18], 3 months of exposure to a book of monkey faces (starting at 6 months) was sufficient to preserve such recognition abilities at 9 months of age. Scott and Monesson [19] replicated this study with one key difference: Infants learned six monkey faces individually (each face had its own name), categorically (each face was labeled “monkey”), or the faces were not labeled. Only infants trained with individually labeled faces maintained the ability to discriminate the monkey faces at 9 months. Thus, experience individuating faces is essential for infants to benefit from the training.

The convergence of findings from studies on face species [17] and race [1] indicate that 6 to 9 months of age represents an important time of transition in the face processing system. If a certain type of face (other species or race) is not experienced prior to this period, then the ability to discriminate between individual faces within those categories declines. Perceptual training experienced near the end of the tuning period appears to be effective for maintaining the ability to discriminate between individuals of other species. It is possible that such training may also be effective for maintaining the ability to discriminate between other face types, such as other-race faces.

The aim of the current research is to establish whether providing exposure to other-race faces between 6 and 9 months of age will change infants' abilities to process other-race faces. This work will determine whether exposure to other-race (Chinese) faces through perceptual training via picture books allows infants to (1) learn the individual faces from the books, and (2) maintain the ability to discriminate individual Chinese faces at 9 months of age. Performance will be assessed using the paradigm previously used to investigate infants' abilities to learn and discriminate individual monkey faces [18]. We hypothesized that 9-month-old Caucasians who received long-term book exposure to Chinese faces would discriminate individual Chinese faces and recognize the exemplars from their books. By contrast, the control group who received training on Caucasian faces would not be able to discriminate Chinese faces at 9 months of age, but would be able to recognize the Caucasian faces from their books.

## Method

### Ethics Statement

This study received ethical approval from the Behavioural & Social Sciences Ethical Review Committee at the University of Queensland prior to commencement of the study.

### Participants

Each infant's parent gave their written informed consent for their child to participate in the study. Sixteen 6-month-old Caucasians (8 girls; mean age  = 185 days, range  = 175 to 194 days) were included in the training group. Two infants were excluded because of crying/fussing during the pre-training test. Two infants were excluded because they were unable to return for testing at 9 months. One infant was excluded due to experience with Asians, as the result of a Japanese student living in their home. The training group infants included for analyses returned at 9 months (mean age  = 276 days, range  = 260 to 284 days).

Sixteen 6-month-old Caucasians (9 girls; mean age  = 186 days, range  = 176 to 194 days) were included in the control group. Two additional infants were excluded due to crying or fussing, and another two were excluded because they did not return for testing at 9 months. The control group infants included for analyses returned at 9 months (mean age  = 274 days, range  = 258 to 284 days). Parents' responses on a questionnaire indicated that all infants had minimal contact with other-race individuals. Infants were tested in Brisbane, Australia.

### Stimuli

**Figure 1:**
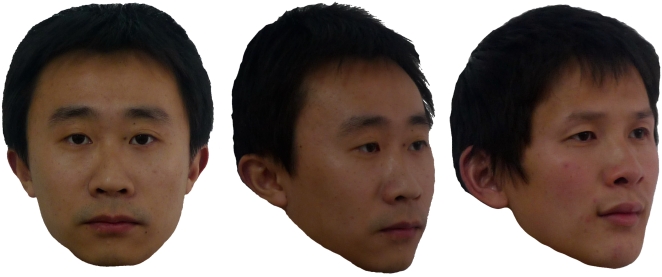
Examples of Chinese face exemplars presented to infants. The first face is presented during the familiarization phase and the second and third faces are presented as the discrimination test. These particular face exemplars were not used in the study and are used for illustrative purposes only, however they are depicted in exactly the same manner as the study stimuli.

The stimuli used in the training books were 48 color pictures: 24 Chinese faces in the training group and 24 Caucasian faces in the control group, all presented against a white background (Fig. 1). For each race, half of the faces were female. There were eight different training books each measuring 22×30 cm. The books contained either six female faces or six male faces (with names) and were either all Caucasian or all Chinese, resulting in the following series of books: two containing Chinese males, two with Chinese females, two with Caucasian males, and two with Caucasian females. Faces were presented in frontal orientations and with neutral expressions. Pictures were cropped so that hairlines were visible, but hairstyles were fairly uniform across both the male and female stimuli. The gender of the face stimuli for the 6-month discrimination task, training protocol, 9-month retention task, and 9-month discrimination task was consistent for each infant.

A new set of faces was used for the VPC test assessing the infant's ability to discriminate between novel Chinese faces (see Fig 1). Half of the infants were presented with a front-facing image during familiarization and two three-quarter pose faces for the test trials. The other half were presented with the reverse. Thus, two different Chinese faces were needed for each discrimination test, and one of these faces was presented in a different orientation (3 images in total). Six different face sets (each containing 3 images) served as the VPC stimuli: 3 Chinese male and 3 Chinese female sets. Stimulus size and brightness were kept uniform by using Adobe Photoshop. When projected onto the screen, each picture was 15 cm high and 10 cm wide. Only one stimulus was projected in the centre of the screen for familiarization, and two stimuli were projected side by side separated by a 12-cm gap during the retention tests.

### Procedure

A VPC procedure [20], [21] was used to assess facial discrimination in infants before (at 6 months) and after an exposure/training period with Chinese faces (at 9 months). The VPC procedure indexes the level of interest for one stimulus in a pair after one of these stimuli has been learned during a prior familiarization period. Recognition is inferred from the infant's tendency to fixate on one stimulus, generally the novel one, for a longer duration. A Visual Preference task was also utilized to assess recognition of individual faces from the infants' specific book at 9 months of age. A systematic preference for one face (novel or familiar) indicates recognition.

Testing took place in a quiet room. Infants were seated in a high chair with their mother beside them. Images were projected onto a large screen in front of the infant. A digital camera filmed the infant's eye movements.

#### Pre-training testing

At 6 months, the infant's pre-training ability to discriminate between either individual Chinese or Caucasian faces was assessed with a VPC task. Infants were familiarized to a Chinese or Caucasian face for 20 s of cumulative looking. Stimulus fixation was assessed by the corneal reflection technique [22]. An experimenter hidden from the infant's view examined the eyes on the camera screen and controlled the time of presentation of stimuli during the familiarization, inter-trial intervals, and preference tests. A computer algorithm determined when 20 s of cumulative looking time was reached. The discrimination tests started when the infant looked at one of the two stimuli, and they ended after 5 s had elapsed. After the first 5-s test, the side on which the images were presented was reversed, and a second 5-s test was completed.

#### Training

After the pre-training session, parents were provided with a book containing images of either Chinese (other-race) or Caucasian (own-race) faces. The images were different from those used during the pre-training test. Parents were asked to present the pictures in the book to their child for 2–3 minutes every day for 1 week, then every other day for the next week, and then less frequently (approximately once every 6 days) following a fixed schedule of exposures during the 3-month period (equating to approximately 70 minutes of exposure overall). This is based on the schedule used to successfully train 6-month-old infants on monkey faces [18], [19]. Parents were supplied with detailed instructions about the training schedule. Parents were contacted via phone every 3–4 weeks to ensure compliance with the book training protocol.

#### Post-training Testing

*Recognition of trained exemplars:* Three months later, 9-month-olds' abilities to recognize pictures from the training book was assessed. There were six recognition trials, one for each face included in the infant's training book. For each trial, a familiar image from the book was presented with a novel image until 10 s of cumulative looking was recorded. For half of the trials, the familiar image was the exact image from the training book (i.e. facing front). For the other half, a three-quarter pose of the same person was presented. Varying the viewpoint between learning and test ensures that infants are actually learning the face, rather than just the picture. Each trial was followed by a blank screen for 2 s. The pairing of the pictures was completed by the experimenters on the basis of pictures being similar but distinguishable. The position of the familiar pictures was counterbalanced across trials to control for side preference. The six faces which were familiar for one group served as novel for the other group and vice versa. This controlled for the possibility that some faces were more attractive for reasons other than familiarity.

*Generalization to novel exemplars:* After the recognition test and a short break, a post-training VPC test was conducted in the same way as the pre-training task (using a new set of Chinese faces) to assess the infant's ability to discriminate between novel Chinese faces. Infants were exposed to a Chinese face during the familiarization phase and then tested with the familiar and novel Chinese face. Orientation was counterbalanced across infants so that half the infants within each condition were familiarized to the three-quarter pose face and tested on front-facing pictures, and the other half of the infants were presented with the reverse. This procedure established whether the training facilitates generalization of other-race face discrimination skills (i.e., general learning about race has occurred), or whether infants are simply remembering individual faces. Each pair of photographs was presented for 5 s. There were two test conditions: (1) Learn Chinese faces, test on Chinese faces (training group) and (2) Learn Caucasian faces, test on Chinese faces (control group).

#### Reliability

The time each infant spent looking at stimuli during recognition and generalization tests was examined using frame-by-frame analysis. Testing sessions were analyzed by two observers blind to the location of the novel and familiar test stimuli. Inter-observer reliability was calculated for 20% of the infants. The six retention trials, two discrimination test trials, and the time required to reach familiarization criteria were double-scored for each of these infants. Direction of looking was compared for each 40-ms frame. The average level of agreement was 95%.

## Results

For each of the analyses, preliminary examination of the data revealed no significant differences due to gender or orientation of the face photographs, so the data were combined for further analysis.

### Pre-training Discrimination Test: 6 months

A 2 (Condition: Chinese vs. Caucasian) x 2 (Face stimulus: Novel vs. Familiar) mixed model ANOVA was conducted on total looking time to the stimuli. This analysis revealed a significant main effect of Condition, *F*(1,30)  = 5.67, *p = *.023, η^2^ = .15, indicating that 6-month-olds looked longer at Chinese than Caucasian faces (4.76 s or 52% vs. 4.39 s or 48%, respectively), averaged across the novel and familiar categories. This indicates that infants looked longer at the pair of Chinese faces on the test trial, following familiarization to a Chinese face. There was also a significant main effect of Stimulus, *F*(1,30)  = 20.11, *p = * <.001, η^2^ = .39; however, the Stimulus x Condition interaction was not significant, *F*(1,30)  =  <1, *p = *.994, η^2^ = .01. This result indicates that 6-month-olds look significantly longer at the novel than familiar face stimuli (5.39 s or 59% vs. 3.76 s or 41%, respectively) averaged across both the Caucasian and Chinese conditions. Thus, 6-month-olds discriminated the individual Chinese or Caucasian faces without training (see Table 1), thus replicating the findings of Kelly et al. [1].

**Table 1:** Mean looking times (s), corresponding percentage of looking time and standard deviations (in parentheses) for novel and familiar faces for each condition for the 6-month-olds prior to training.

Stimuli	Novellooking time	Novel%	Familiar looking time	Familiar%
Chinese	5.57 (1.03)	59	3.94 (1.02)	41**
Caucasian	5.21 (1.32)	59	3.57 (1.22)	41**

**p<.01.

### Recognition of Trained Exemplars: 9 Months

Looking times for the six novel faces were summed as were looking times for the six familiar faces for both the Chinese and Caucasian conditions. A 2 (Face stimulus: Novel vs. Familiar) x 2 (Condition: Chinese vs. Caucasian book training) mixed model ANOVA was conducted on total looking time to the stimuli. The main effect of Stimulus was not significant, *F*(1,30) <1, *p = *.716, η^2^ = .005, and the main effect of Condition was marginally significant, *F*(1,30)  = 3.35, *p* = .08, η^2^ = .10, but this was subsumed by a significant Stimulus x Condition interaction, *F*(1,30)  = 14.42, *p* = .001, η^2^ = .33. Follow-up paired-samples two-tailed *t*-tests indicated that infants looked longer at the novel than familiar Caucasian faces during retention test trials if they received Caucasian face training, *t*(15)  = 2.33, *p* = .034 (see Table 2). Infants who received Chinese face training looked significantly longer at the familiar than novel Chinese faces, *t*(15)  = 3.11, *p* = .008 (see Table 2).

**Table 2:** Mean looking times (s), corresponding percentage of looking time and standard deviations (in parentheses) for novel and familiar faces from Caucasian and Chinese training books for the 9-month-olds following training.

Book training	Novellooking time	Novel%	Familiar looking time	Familiar%
Chinese	27.58 (3.24)	46	32.42 (3.16)	54**
Caucasian	31.93 (3.52)	53	28.07 (3.00)	47*

*p<.05; **p<.01.

This systematic preference for one category (i.e., familiar or novel) demonstrates that infants learned and remembered the faces from their picture books. However, the preference for the novel or familiar face on retention test trials differed depending on the race of the face. Nine-month-olds who received Caucasian face training looked longer at the *novel* than familiar Caucasian faces during test trials. In contrast, infants who received Chinese face training looked longer at the *familiar* than novel Chinese faces. This difference in preference depending on the trained race may reflect the different levels of complexity/difficulty involved in processing own- versus other-race stimuli. Chinese faces are presumably more difficult for young Caucasian infants to process and recognize/remember due to limited experience with this race (even after training). Infants tend to show a preference for familiar stimuli when the task is complex [23]. Additionally, infants may require more time to recognize an other-race face as familiar, resulting in longer looking at the familiar category for other-race faces. In contrast, Caucasian faces are easier to process and recognize since Caucasian 9-month-olds have a wealth of experience with Caucasian adult faces. Caucasian 9-month-olds could rapidly recognize individual Caucasian faces and then shift their preference to look at something new (the novel face), thereby demonstrating recognition of the familiar face stimuli via novelty preference.

### Generalization to Novel Exemplars: 9 Months

A 2 (Condition: Chinese vs. Caucasian book training) x 2 (Face stimulus: Novel vs. Familiar) mixed model ANOVA was conducted on total looking time to the stimuli. There was no main effect of condition, *F*(1,30)  =  <1, *p = *.775, η^2^ = .003. There was a significant main effect of Stimulus, *F*(1,30)  = 4.64, *p = *.04, η^2^ = .14, which was subsumed by a marginally significant Stimulus x Condition interaction, *F*(1,30)  = 3.08, *p = *.08, η^2^ = .10.

Follow-up paired-samples two-tailed *t*-tests conducted for infants who had received Chinese face training revealed that these 9-month-olds looked significantly longer at the novel than familiar Chinese faces, *t*(15)  = 3.32, *p* = .007 (see Table 3). This result indicates that after 3 months of exposure with Chinese faces, Caucasian 9-month-olds can discriminate individual exemplars from this race. Follow-up paired-samples two-tailed *t*-tests conducted for the Caucasian book training/control condition (i.e., 3 months of training on Caucasian faces; tested for individual recognition of Chinese faces) indicated no significant difference in looking times for the novel and familiar Chinese faces, *t*(15) <1, *p* = .801. Without training on Chinese faces, Caucasian 9-month-olds cannot discriminate individual Chinese faces, thereby replicating the results of Kelly et al. [1].

**Table 3:** Mean looking times (s), corresponding percentage of looking time and standard deviations (in parentheses) for novel and familiar faces for each condition for the 9-month-olds following training.

Book training	Test Stimuli	Novellooking time	Novel%	Familiar looking time	Familiar%
Chinese	Chinese	5.26 (1.00)	57	3.96 (0.83)	43**
Caucasian	Chinese	4.73 (1.21)	51	4.60 (1.10)	49

**p<.01.

## Discussion

The studies reported examined the effect of exposure to Chinese faces on the specialization of the face-processing system to own-race faces during the first 9 months for Caucasian infants. We investigated whether providing Caucasian 6-month-olds with perceptual training on Chinese faces via picture books allows them to learn the individual pictures in their books and maintain the ability to discriminate Chinese faces at 9 months. This is an ability that otherwise is lost due to tuning of the human face prototype.

The results indicate that infants can remember and recognize individual faces they have seen in a picture book across a 3-month period, for both own- and other-race faces. Infants generalized from the front-facing pictures in their book to recognize the same person in a different orientation (three-quarter pose) during test trials. This finding indicates that infants were actually learning the face, rather than just the picture.

Next, we investigated whether Caucasian 9-month-olds who received Chinese face training could generalize their knowledge to novel Chinese face exemplars. This study is the first to demonstrate that exposure to Chinese faces from 6 to 9 months of age is sufficient to maintain Caucasian infants' abilities to discriminate Chinese faces. Three months of exposure to 6 individual other-race faces in a picture book prevents tuning of the face processing system towards just one race of face. Consistent with Kelly et al. [1], we found that Caucasian 9-month-olds who did not receive exposure to Chinese faces could not discriminate individual Chinese faces. These results are in line with the findings that infants' preferences for faces of different races are dependent on their experience with faces of a given race [4], [5], [6] and Nelson's [3] hypothesis that the face prototype develops according to the type of facial input received. Our findings also support previous research demonstrating that although the face-processing system becomes tuned to own-race faces very early in life, it still retains flexibility to process other-race faces, given sufficient exposure [10], [11]. Finally, our results are consistent with previous research demonstrating that 6 to 9 months of age is an important period for tuning of the face prototype towards own-species and own-race faces [1], [17].

Future research should establish the constraints on learning about other-race faces. This includes the duration of training necessary to discriminate other-race faces, the age point when the training must begin, and the age limit where training would be effective. The next step is to investigate the duration of the training effect, i.e., for how long after the training ceased would Caucasian infants maintain the ability to discriminate individual Chinese faces? Finally, does the face training need to be specific to the other-race faces that will be tested for individual recognition at 9 months of age? Or could training with Chinese faces improve performance with African faces?

In conclusion, this study demonstrates that perceptual training via picture books is effective in producing a change in the face processing system at 9 months when the stimuli are other-race faces. Previous research has demonstrated that the face representation for race information is malleable in children and adults if the appropriate input or training is available. The present study is unique in demonstrating that training on other-race faces can prevent the ORE from developing in the first place.
